# Multispectral remote sensing for accurate acquisition of rice phenotypes: Impacts of radiometric calibration and unmanned aerial vehicle flying altitudes

**DOI:** 10.3389/fpls.2022.958106

**Published:** 2022-08-10

**Authors:** Shanjun Luo, Xueqin Jiang, Kaili Yang, Yuanjin Li, Shenghui Fang

**Affiliations:** ^1^School of Remote Sensing and Information Engineering, Wuhan University, Wuhan, China; ^2^Lab for Remote Sensing of Crop Phenotyping, Wuhan University, Wuhan, China

**Keywords:** multispectral remote sensing, unmanned aerial vehicle (UAV), radiometric calibration, UAV flying altitude, precision agriculture

## Abstract

As a promising method, unmanned aerial vehicle (UAV) multispectral remote sensing (RS) has been extensively studied in precision agriculture. However, there are numerous problems to be solved in the data acquisition and processing, which limit its application. In this study, the Micro-MCA12 camera was used to obtain images at different altitudes. The piecewise empirical line (PEL) method suitable for predicting the reflectance of different ground objects was proposed to accurately acquire the reflectance of multi-altitude images by comparing the performance of the conventional methods. Several commonly utilized vegetation indices (VIs) were computed to estimate the rice growth parameters and yield. Then the rice growth monitoring and yield prediction were implemented to verify and evaluate the effects of radiometric calibration methods (RCMs) and UAV flying altitudes (UAV-FAs). The results show that the variation trends of reflectance and VIs are significantly different due to the change in component proportion observed at different altitudes. Except for the milking stage, the reflectance and VIs in other periods fluctuated greatly in the first 100 m and remained stable thereafter. This phenomenon was determined by the field of view of the sensor and the characteristic of the ground object. The selection of an appropriate calibration method was essential as a result of the marked differences in the rice phenotypes estimation accuracy based on different RCMs. There were pronounced differences in the accuracy of rice growth monitoring and yield estimation based on the 50 and 100 m-based variables, and the altitudes above 100 m had no notable effect on the results. This study can provide a reference for the application of UAV RS technology in precision agriculture and the accurate acquisition of crop phenotypes.

## Introduction

The precise, economical, and dynamic gathering of farmland information in time is critical for boosting agricultural economic development and reducing labor costs (Wang et al., 2021). Rice (*Oryza sativa* L.) being a significant global staple food crop, its growth status and yield level have always been of great concern (Wan et al., 2020; Duan et al., 2021). Exact and pre-harvest growth monitoring and yield prediction of rice is of much value for the implementation of field measures, policy formulation, and price judgment (Zheng et al., 2019; Wan et al., 2020).

Manual investigation and measurement in the fields are frequently used methods for collecting rice yield and phenotypic data. This includes labor- and time-intensive work with great uncertainty (Yang et al., 2017). Furthermore, some phenotypic parameters can only be gained by destroying the samplings, resulting in the interruption in obtaining growth parameters in the later periods (Zhao et al., 2021). Rice yield formation is a process of dynamic accumulation, which is associated with both vegetative and reproductive growth periods (Wan et al., 2020). Therefore, it is essential to continuously monitor the growth status of rice (Yue et al., 2019).

In contrast, the prominent merit of remote sensing (RS) is that a large area can be covered and the phenotype information can be obtained in a non-destructive way (Franch et al., 2021). The development of the RS technique makes it possible to forecast the crop yield in advance. At present, the prediction accuracy of satellite RS has reached a relatively high level (Li et al., 2014; Huang et al., 2015; Franch et al., 2021). However, the images derived from satellites generally have some insurmountable shortcomings. For example, the resolution is rarely up to centimeter level, and the occlusion of clouds is serious. Hence, the flexibility of getting plant phenotype information on time is limited. Moreover, the scale of satellite RS cannot meet the practical application needs in precision agriculture like the small plots in southern China (Peng et al., 2019). In recent years, unmanned aerial vehicle (UAV) RS technology has become a hot content in the field of agricultural research. The UAV provides a convenient tool for emerging sensors to obtain unparalleled images with high time–space–spectral resolution (Maes and Steppe, 2019).

The low-altitude UAV-based RS technology is of high significance to precision agriculture owing to its high efficiency, low cost, and macro size (Deng et al., 2018b; Han et al., 2019). Different types of sensors have also been applied to different scenes. For example, hyperspectral cameras were exploited to estimate crop leaf area index (LAI), leaf chlorophyll content, and leaf nitrogen content (Delegido et al., 2010; Xie et al., 2014; Raj et al., 2021). Thermal infrared sensors were utilized to retrieve the temperature and water status of the olive tree canopy (Noguera et al., 2020) and help to predict the soybean yield (Maimaitijiang et al., 2020). Moreover, the UAV-based LiDAR sensors were recently employed to determine the height of different crops (ten Harkel et al., 2020). Although they showed unique performance in precision agriculture, there are some difficulties in practical use. On the one hand, for the current UAVs, the weights of the sensors exceed the standard ones, which makes the flight difficult to last for a long time. On the other hand, these sensors are very expensive, and few people can afford the high cost. As non-quantitative RS equipment, the visible sensor (RGB camera) based on broadband is often used in precision agriculture due to its acceptable price and convenient operation. However, on account of the limitation of the number of bands and difficulty in calibration, it is generally applied to provide canopy height information and describe color changes (Deng et al., 2018b). Multispectral sensors cannot only meet the cost requirements but can also obtain high-resolution multispectral images, including red-edge and near-infrared (NIR) bands sensitive to vegetation growth (Huang et al., 2018). Therefore, the multispectral camera has great potential in precision agriculture. Vegetation indices (VIs) based on multispectral images have been extensively used in crop growth monitoring and yield prediction (Deng et al., 2018b; Gong et al., 2018; Duan et al., 2019).

For multispectral sensors, the premise of extracting accurate RS phenotype information is to obtain high-precision canopy spectral reflectance data. The observed reflectance data of the same ground object by different multispectral sensors are often different. The main reasons are as follows: (i) equipment and methods used for radiometric calibration (without considering the influence of the atmosphere), (ii) observation geometry, and (iii) differences in spectral response functions of different sensors in the corresponding bands (Deng et al., 2018a,b). When the multispectral sensor, observation target, and time are determined, the main factors affecting the observed reflectance are radiometric calibration methods (RCMs) and observation angles. In practical application, UAV control is flexible and changeable, resulting in flight altitudes ranging from dozens to hundreds of meters. Different flight altitudes will not only lead to changes in RS image resolution but also to differences in the observation angles of the same target. Currently, there are few reports on this aspect. Therefore, the impacts of UAV flying altitudes (UAV-FAs) on multispectral data are worth discussing.

In this study, the multispectral images derived from different UAV-FAs in some experiments (including different types of ground objects, different fertilizer gradients, and multi-cultivar rice experiments) were obtained to (i) compare the accuracy of reflectance and VIs achieved by different RCMs and put forward a unified calibration method suitable for the accurate acquisition of reflectance of different objects, (ii) analyze the variation trend and causes of reflectance and VIs at different UAV-FAs, and (iii) evaluate the effects of different RCMs and UAV-FAs on rice growth monitoring and yield prediction.

## Materials and methods

### Experimental area

This study involved three experimental areas, including Ezhou City, Hubei Province (30°22′31″N,114°44′50″E), Lingshui County, Hainan Province (18°31′47″N, 110°03′35″E), and Wuhan University Friendship Square, Hubei Province (30°31′48″N, 114°21′20″E), China.

Four experiments (named Tests 1, 2, 3, and 4) were conducted in this study. Test 1 was carried out from February to April 2018. A total of 42 rice plots including 42 cultivars were set up, and each plot covered an area of approximately 40 m^2^. Except for different rice cultivars, the fertilization level and drainage and irrigation management were the same. For more details, see Jiang et al. (2019). Test 2 included three repetitions and four nitrogen gradients (0, 120, 180, and 240 kg/ha) and was conducted from February to April 2018. Two rice cultivars and 24 plots were randomly arranged, of which each plot was about 30 m^2^. Black plastic film was laid between plots with different gradients to isolate water and fertilizer. Test 3 was the small rice plot experiment with an area of 1 m^2^ of each plot and was conducted from July to September 2018. Multiple plots were selected to conduct the multi-altitude experiments. One rice cultivar was planted in each plot at an interval of 10 cm. In addition to their respective control variables (cultivar and nitrogen fertilizer), other field management measures (irrigation, weeding, pesticide application, etc.) were implemented under the guidance of professionals. The main purpose of Test 4 was to study some ground objects different from rice (grassland, smooth, and rough slabstone) for comparative analysis and to increase the universality of the results. We randomly selected and marked 17 locations, including grassland and slabstone, as the research objects on 23 July 2021.

### Field data acquisition

In Test 1 and Test 2, rice growth parameters including LAI, above-ground biomass (AGB), and canopy chlorophyll content (CCC) at different growth durations (tillering, jointing, booting, heading, and milking stages) were measured directly or calculated indirectly.

The LAI (unitless) was measured by using LAI-2200C (LI-COR, Lincoln, Nebraska United States) at dusk or dawn. Three repeated measurements were conducted for each rice plot, and the mean value was treated as the plot-level LAI. Each repetition result is a reading of 10 random measurements (1 above and 10 below the rice canopy) with a 270° view cap. The LAI value obtained in this way can be regarded as the green LAI (LAI_green_) due to the strong correlation with the manually measured value (*R*^2^ = 0.87, *P* < 0.001) (Liu et al., 2017).

The SPAD (unitless) values, which were often used to reflect the chlorophyll content level of leaves (Uddling et al., 2007), were measured using SPAD-502 meter (Spetrum Technologies, Inc., Plainfield, IL, United States). At each growth stage of rice, 10 plants in every plot were selected to measure the SPAD values of four upper fully expanded leaves (SPAD_upper_) at multi-locations (Peng et al., 1993), and the average was treated as the plot-level SPAD.

AGB (in g/m^2^) was collected by destructive sampling. Three hills of plants were dug and taken back to the laboratory for drying. All samples with the underground parts removed were dried at 80°C until multiple weighing values remained constant. The plot-level AGB was the product of dry weight and density of samplings.

The CCC (in g/m^2^) was calculated by exploiting the product of LAI_green_ and SPAD_upper_ (Baret et al., 2007) to diagnose the canopy nitrogen content (Gitelson et al., 2005; Liu et al., 2017) and assess the total canopy-scale productivity of rice (Inoue et al., 2016).

The rice yield (in g/m^2^) was surveyed by sampling 100 plants in each plot. The final rice yield of the plot was the average weight of spikes after threshing and drying multiplied by the transplanting density.

At different stages, the ASD Field Spec 4 spectrometer (Analytical Spectral Devices Inc., Boulder, CO, United States) was adopted to collect rice canopy spectra under cloudless and windless conditions. The multiple and multipoint measurements were implemented daily from 10:00 to 14:00 with a field-of-view (FOV) of 25°, and the results of five points and 10 measurements at each point were averaged to get the plot-level spectra.

### Unmanned aerial vehicle multispectral image collection

The Micro-MCA camera fixed on the UAV (Duan et al., 2021) with a gimbal was utilized to obtain the multispectral images from 11:00 to 13:00. Twelve independent camera lenses (the image size of 1,280 × 1,024 pixels, with the horizontal and vertical FOV of 38.26 and 30.97°) were equipped with central bands of 490–950 nm. The visible to NIR bands widely employed in precision agriculture were covered (Kimes et al., 1981). The details of UAV multispectral data acquisition are shown in Table 1.

**TABLE 1:** The details of multispectral images for four experiments in Hubei and Hainan Provinces.

Experimental name	Study site	Number of plots	Date of UAV images collection (DAT/day)	UAV flying altitude (m)	Growth stage of rice
Test 1	Hainan	42	2018/02/02 (25)	100, 150, 200, 250	Tillering stage
			2018/02/26 (49)		Jointing stage
			2018/03/11 (62)		
			2018/03/18 (69)		Booting stage
			2018/04/01 (83)		Heading stage
			2018/04/26 (108)		Milking stage
Test 2	Hainan	24	2018/02/02 (27)	50, 100	Tillering stage
			2018/02/20 (45)		
			2018/03/03 (56)		Jointing stage
			2018/03/11 (64)		
			2018/03/25 (78)		Booting stage
			2018/04/01 (85)		Heading stage
			2018/04/26 (110)		Milking stage
Test 3	Hubei	Multiple	2018/08/15 (52)	60, 70, 80, 90, 100, 110, 120, 130, 150, 170, 190, 210, 230, 250	Tillering stage Jointing stage Booting stage Heading stage Milking stage
Test 4	Hubei	17	2021/07/23	60, 70, 80, 90, 100, 110, 120, 130, 150, 170, 190, 210	−

### Data processing

The noise, vignetting, distortion correction, and band-to-band alignment of multispectral images were determined in the PixelWrench 2 software (Xu et al., 2019). Subsequently, three RCMs were applied to compare the reflectance conversion accuracy from the original digital number (DN) values. In this process, eight calibration targets (covering different reflectance ranges: 3, 6, 12, 24, 36, 48, 56, and 80%) with stable reflectance were laid within the imaging range of the UAV to obtain the reflectance of the target ground object. The eight 1.2 × 1.2 m grayscale calibration panels were fabricated using a specific lot of coated fabric for Tetracam Inc.

The conventional empirical line (EL) method was extensively employed when only two calibration targets were used for its straightforward, simple, and effective implementation (Smith and Milton, 1999; Laliberte et al., 2011; Wang and Myint, 2015). Since the fact that the increased number of observation targets could improve the accuracy and reliability of calibration was proved (Tucker, 1979), multiple calibration panels were utilized to obtain the reflectance by the EL method (Xu and Huang, 2008; Duan et al., 2019, 2021; Wan et al., 2020). However, in many vegetation scenes, it was found that the calibrated reflectance values of visible bands were negative, especially in blue and red bands (Deng et al., 2018b). In practice, it is worth noting that the linear relationship between reflectance and radiance does not always exist (Stow et al., 1996). Therefore, non-linear models need to be considered during the radiometric calibration. The subband empirical line (SEL) method was proposed to solve the problem of negative reflectance (Deng et al., 2018a,b). In the SEL method, different bands were divided into two groups (red, green, and blue bands with low reflectance and red edge and NIR bands with high reflectance), and the power and linear models were used for the reflectance calibration, respectively. The SEL method only studied the scene of vegetation’s low reflectance in visible bands and did not consider other objects with high reflectance in these spectral regions. Moreover, the case of multiple calibration panels was ignored in the SEL method. In addition, the comparison of the linear and power models in visible bands with high reflectance was not performed. In this study, the piecewise empirical line (PEL) method was put forward to construct a general calibration method for reflectance acquisition of different types of objects. In all experiments, images obtained at a height of 100 m were selected for radiometric calibration comparison. The transformation relations of these three RCMs are shown in Eqs. (1–3).


(1) $$y=a_{1}\cdot x_{i}+b_{1}$$

(2) $$y=\left\{ \begin{array}{c} a_{2}\cdot x_{i}^{b_{2}}\;i=1,2,3,4,5,6,7\\ a_{3}\cdot x_{i}+b_{3}\;i=8,9,10,11,12 \end{array}\right.$$

(3) $$y=\left\{ \begin{array}{c} a_{4}\cdot x_{i}\;y\leq 0.03\\ a_{5}\cdot x_{i}+b_{5}\;y>0.03 \end{array}\right.$$

where y is the reflectance after radiometric calibration, x is the DN value of different bands, i is the number of bands from 1 to 12, and the constants a and b are the corresponding slopes and intercepts of the fitted lines from the used calibration targets.

To compare the accuracy of the measured spectra using the ASD spectrometer (R_ASD_) with the UAV multispectral reflectance (R_MCA_), the R_ASD_ needs to be convolved by the spectral response function of the MCA camera (MCA-based equivalent reflectance, R_MCA–ASD_). The conversion of spectral reflectance was calculated by Eq. (4).


(4) $$R_{j}=\frac{\int_{s}^{e}R_{\mathrm{ASD}}\cdot S_{j}\left(\lambda \right)d\text{λ}}{\int_{s}^{e}S_{j}\left(\lambda \right)d\text{λ}}$$

where R_*j*_ is the R_MCA–ASD_, λ is the wavelength, S_*j*_(λ) is the spectral response function of band j, and e and s are the starting and ending wavelengths of band j, respectively.

The plot-level canopy reflectance of rice derived from UAV images was obtained by defining a rectangular region of interest (ROI). For different UAV-FAs, the reflectance was acquired by adjusting the number of pixels in the ROI of the responding image according to the resolution. Additionally, several VIs, such as RVI (Jordan, 1969), NDVI (Tucker, 1979), NDRE (Gitelson and Merzlyak, 1994), VARI (Gitelson et al., 2002), EVI2 (Jiang et al., 2008), CI_*rededge*_ (Gitelson et al., 2003), CI_green_ (Gitelson et al., 2003), MCARI (Daughtry et al., 2000), and WDRVI (Gitelson, 2004), frequently applied to rice growth monitoring and yield prediction were computed.

### Methods and evaluation

In practical multispectral images, each pixel is usually a mixed pixel. The fluctuation in the reflectance of the mixed pixel can be regarded as the changes in the components (called endmember) and proportions of these components (called abundance). Mixed pixel decomposition is a process of calculating the abundance of each component by using the least-square method under the condition that the reflectance of the mixed pixel and each endmember is known. The fully constrained least-square linear spectral mixture (FCLS-LSM) model was employed in this study to obtain the abundance of endmembers of multispectral images derived from different UAV-FAs with the constrained conditions of Eqs. (5–6) (Gong et al., 2018; Duan et al., 2019).


(5) $$R=\sum_{i=1}^{n}A_{i}R_{i}+e$$

(6) $$0\leq A_{i}\leq 1;\sum_{i=1}^{n}A_{i}=1$$

where R is the reflectance of the mixed pixel, A_*i*_ is the abundance of the endmember i, R_*i*_ is the reflectance of the endmember i, n is the number of the endmembers, and e is the error.

The prediction models of rice growth parameters and yield were constructed using linear regression and three machine learning algorithms of SVR, RFR, and ANN (Ashapure et al., 2020). The accuracy was described quantitatively by *R*^2^, RMSE, and RRMSE (Duan et al., 2021). In addition, the mean relative percent error (MRPE) and RMSE were utilized to analyze and compare the effects of three RCMs. The relevant expressions for calculation are as follows:


(7) $$MRPE=\frac{1}{n}\sum_{i=1}^{n}\left|\frac{\hat{y}-y}{y}\times 100\right|$$

(8) $$RMSE=\sqrt{\frac{\sum_{i=1}^{n}{\left(\hat{y}-y\right)}^{2}}{n}}$$

(9) $$RRMSE=\frac{RMSE}{\bar{y}}$$

where y, $\hat{y}$, and $\bar{y}$ are the observed, estimated, and measured mean values, respectively. n is the number of samples. In the RCM evaluation, y is the R_MCA–ASD_.

## Results

### Accuracy comparison of reflectance and vegetation indices based on three radiometric calibration methods

#### The reflectance of rice canopy

**FIGURE 1:**
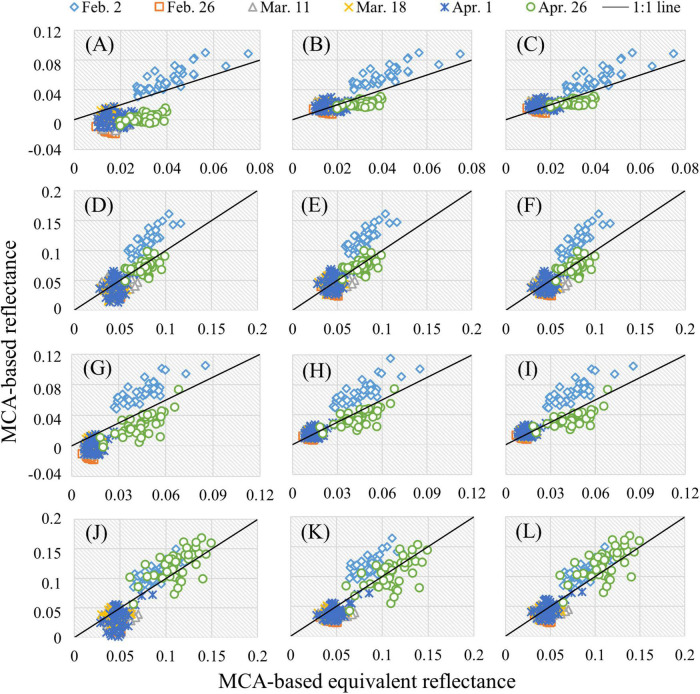
Comparison of R_MCA_ and R_MCA–ASD_ in Test 1: **(A)** 490 nm and EL; **(B)** 490 nm and SEL; **(C)** 490 nm and PEL; **(D)** 550 nm and EL; **(E)** 550 nm and SEL; **(F)** 550 nm and PEL; **(G)** 670 nm and EL; **(H)** 670 nm and SEL; **(I)** 670 nm and PEL; **(J)** 700 nm and EL; **(K)** 700 nm and SEL; and **(L)** 700 nm and PEL.

The R_MCA–ASD_ of rice canopy during the whole growth period in Test 1 and Test 2 was used to compare with the R_MCA_ based on three RCMs (EL, SEL, and PEL). Since the differences among RCMs mainly lie in the visible and red-edge (700 nm) bands, these bands will be discussed emphatically. The accuracy comparison of several typical bands (490, 550, 670, and 700 nm) in Test 1 was shown as an example. As can be seen in Figure 1, each row represents a band and each column a RCM. In general, compared with EL, the accuracy of SEL- and PEL-based reflectance was improved at different stages (closer to the 1:1 line), particularly pronounced in the jointing, booting, and heading stages. In these periods, the rice canopy reflectance obtained based on EL was significantly underestimated, and even negative values appeared in the bands of 490 and 670 nm. In addition, the SEL- and PEL-based reflectance showed similarities. Furthermore, it was also found that SEL and PEL had a better ability to predict rice reflectance at the milking stage in the bands with low reflectance (490 and 670 nm), while EL and PEL had higher accuracy at tillering stage in the bands with relative high reflectance (490, 670, and 700 nm).

The MRPE and RMSE were computed to quantitatively evaluate the performance of three RCMs in the reflectance prediction of 12 bands in Test 1 and Test 2. It can be seen from Table 2 that EL has a strong reflectance prediction ability in 720 nm and NIR bands (MPRE < 30% in Test 1 and < 15% in Test 2). In contrast to EL, SEL, and PEL improved the reflectance accuracy of 490, 670, and 680 nm by about 37–66% in Test 1 and 45–61% in Test 2. There is no more than a 5% accuracy difference among the three RCMs in 550 nm. PEL achieved the highest prediction accuracy in each band. Compared with SEL, PEL was slightly improved (less than 5%). Thus, in the estimation of canopy reflectance in the whole growth period of rice, the performance of SEL and PEL was significantly better than that of EL, but the accuracy improvement difference between SEL and PEL was limited.

**TABLE 2:** Statistical results of reflectance accuracy of different RCMs in Test 1 and Test 2.

Band (nm)	MRPE (%)						RMSE					
	EL		SEL		PEL		EL		SEL		PEL	
	Test 1	Test 2	Test 1	Test 2	Test 1	Test 2	Test 1	Test 2	Test 1	Test 2	Test 1	Test 2
490	98.825	76.489	34.306	25.430	**32.287**	**21.616**	0.021	0.019	0.010	0.010	**0.009**	**0.009**
520	50.052	52.625	23.816	27.923	**21.619**	**24.966**	0.021	0.019	0.013	0.014	**0.011**	**0.013**
550	26.362	28.312	25.075	25.223	**22.955**	**24.663**	0.019	0.017	0.020	0.016	**0.018**	**0.016**
570	35.310	42.234	28.813	24.158	**24.423**	**22.241**	0.021	0.019	0.020	0.016	**0.017**	**0.014**
670	99.697	66.812	37.191	23.124	**35.888**	**21.598**	0.020	0.018	0.013	0.014	**0.012**	**0.014**
680	84.679	81.757	38.470	23.502	**37.775**	**20.608**	0.020	0.019	0.014	0.015	**0.013**	**0.014**
700	31.522	37.495	24.926	30.032	**20.724**	**28.137**	0.023	0.023	0.020	0.025	**0.016**	**0.020**
720	18.582	13.865	–	–	–	–	0.033	0.023	–	–	–	–
800	20.010	8.771	–	–	–	–	0.096	0.042	–	–	–	–
850	22.698	8.967	–	–	–	–	0.113	0.043	–	–	–	–
900	20.196	8.463	–	–	–	–	0.102	0.040	–	–	–	–
950	27.343	11.631	–	–	–	–	0.1218	0.046	–	–	–	–

The method with the best performance is represented by bold values.

#### The reflectance of grassland canopy and slabstone

The grasslands and slabstones were selected to obtain the R_MCA_ and R_MCA–ASD_ simultaneously to evaluate the performance of the three RCMs in the reflectance predictions of different ground objects. The prediction accuracy of EL and PEL is identical for the observed slabstones due to the fact that the reflectance of each band is not less than 3%. It could be found in Table 3 that EL had a good prediction effect on the reflectance of grassland canopy and slabstone at 720 nm and NIR bands. For grassland, in the low reflectance bands (490, 670, and 680 nm), the performance of PEL was consistent with that of rice. The reflectance accuracy of PEL was significantly higher than that of EL and slightly stronger than that of SEL. However, in the bands of 550, 570, and 700 nm, the results of EL and PEL were similar and significantly better than SEL. For slabstone, EL (PEL) performed better than SEL in each compared band.

**TABLE 3:** Statistical results of reflectance accuracy of different RCMs in Test 4.

Band (nm)	MRPE (%)					RMSE				
	EL		SEL		PEL	EL		SEL		PEL
	Grassland	Slabstone	Grassland	Slabstone	Grassland	Grassland	Slabstone	Grassland	Slabstone	Grassland
490	69.603	**29.806**	44.919	36.096	**42.607**	0.032	**0.043**	0.021	0.053	**0.020**
520	72.858	**35.562**	56.752	42.594	**51.435**	0.052	**0.053**	0.041	0.064	**0.037**
550	24.709	**24.070**	32.618	30.082	**24.709**	0.024	**0.043**	0.032	0.053	**0.024**
570	34.446	**25.030**	35.849	31.071	**34.446**	0.030	**0.043**	0.031	0.05	**0.030**
670	50.253	**24.224**	42.061	29.463	**41.636**	0.029	**0.049**	0.025	0.059	**0.025**
680	64.741	**26.298**	48.397	31.021	**43.571**	0.040	**0.052**	0.030	0.063	**0.027**
700	32.783	**23.615**	38.850	29.056	**32.783**	0.038	**0.050**	0.045	0.063	**0.038**
720	6.630	20.1980	–	–	–	0.016	0.044	–	–	–
800	4.390	17.735	–	–	–	0.017	0.045	–	–	–
850	3.354	17.295	–	–	–	0.014	0.047	–	–	–
900	5.445	16.326	–	–	–	0.025	0.046	–	–	–
950	5.034	24.287	–	–	–	0.022	0.074	–	–	–

The method with the best performance is represented by bold values.

#### Vegetation indices of rice and grassland canopy

The VIs calculated based on R_MCA_ and R_MCA–ASD_ of rice and grassland were compared to analyze the influence of RCMs. Here, only the accuracy comparison of RVI, NDVI, VARI, EVI2, CI_green_, MCARI, and WDRVI was analyzed because the red-edge indices (NDRE and CI_*rededge*_) were a combination of 720 nm and NIR bands, and the comparison of the three RCMs was not involved. The results presented in Table 4 demonstrated that the accuracy of VIs based on the three RCMs was consistent with the accuracy of reflectance. The PEL had the highest accuracy, and the accuracy of SEL and PEL was significantly higher than that of EL. However, CI_green_ in Test 4 showed higher precision of PEL and EL than SEL.

**TABLE 4:** Statistical results of VI accuracy of different radiometric calibration methods in Test 1, Test 2, and Test 4.

Error	Model	Experiment	RVI	NDVI	VARI	EVI2	CI_green_	MCARI	WDRVI
MRPE (%)	EL	Test 1	99.517	4.967	99.321	11.993	42.889	90.532	72.578
		Test 2	99.329	9.860	75.488	11.935	53.042	59.763	90.445
		Test 4	123.900	18.678	84.619	16.780	46.732	36.054	812.925
	SEL	Test 1	31.765	2.091	10.084	9.157	33.601	26.437	41.533
		Test 2	36.040	6.761	43.082	10.999	46.632	23.116	89.540
		Test 4	79.514	15.648	56.319	14.237	67.477	36.914	744.887
	PEL	Test 1	**26.365**	**2.063**	**6.174**	**8.908**	**30.327**	**25.573**	**45.383**
		Test 2	**31.712**	**6.535**	**29.949**	**10.961**	**44.635**	**19.798**	**88.321**
		Test 4	**77.054**	**15.342**	**31.483**	**13.984**	**46.732**	**31.009**	**727.275**
RMSE	EL	Test 1	80.744	0.075	2.490	0.135	5.778	0.022	0.322
		Test 2	78.783	0.090	0.339	0.080	4.505	0.008	0.257
		Test 4	9.663	0.135	0.217	0.089	1.360	0.010	0.347
	SEL	Test 1	13.386	0.040	0.091	0.100	3.460	0.004	0.102
		Test 2	7.529	0.075	0.137	0.075	3.517	0.005	0.156
		Test 4	5.341	0.112	0.133	0.075	1.946	0.010	0.270
	PEL	Test 1	**11.002**	**0.038**	**0.062**	**0.099**	**3.099**	**0.004**	**0.101**
		Test 2	**7.428**	**0.075**	**0.090**	**0.074**	**3.426**	**0.004**	**0.155**
		Test 4	**5.201**	**0.110**	**0.081**	**0.074**	**1.360**	**0.008**	**0.264**

The method with the best performance is represented by bold values.

### Responses of reflectance and vegetation indices to varying UAV flying altitudes

#### The variation of reflectance with UAV flying altitudes

In Test 3, the edge rice plots in the multispectral images at different stages were used to analyze the responses of reflectance and VIs to UAV-FAs. In the same sensor and photographing mode, the resolution of the UAV-induced image was determined by the UAV-FAs. The UAV images of different scales (60–250 m) were converted according to the resolutions, and then the sizes of the ROI were calculated to represent the same study area. The mean value of all pixels within the ROI was taken as the plot-level reflectance.

**FIGURE 2:**
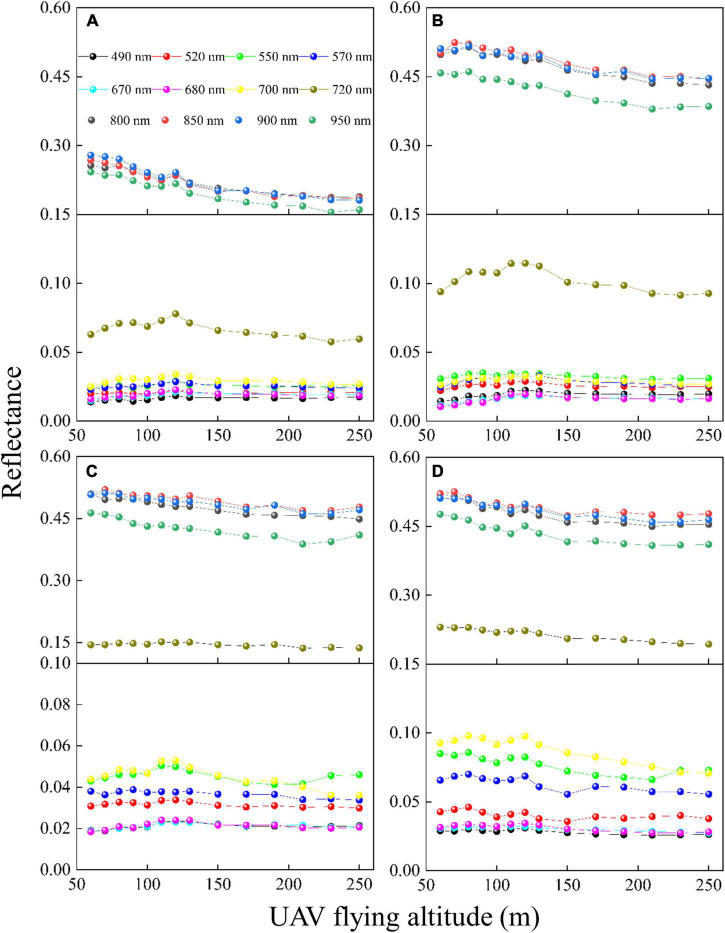
Variation in reflectance of rice with different UAV-FAs at **(A)** tillering stage; **(B)** jointing stage; **(C)** heading stage; and **(D)** milking stage.

It can be found from Figure 2 that the variation trend of rice reflectance (including visible to NIR bands) with UAV-FAs in different periods differs significantly. At the tillering stage (Figure 2A), the reflectance of the NIR bands (800, 850, 900, and 950 nm) decreases obviously with UAV-FAs, but the reflectance of the visible (490, 520, 550, 570, 670, and 680 nm) and red-edge bands (700 and 720 nm) shows a slight rise at first and then a slow decline. At the jointing stage (Figure 2B), in general, the variation trend of reflectance with UAV-FAs in all bands is similar to that at the previous stage, while in the visible and red-edge bands, the reflectance changes more violently within the first 100 m. At the heading stage, the variation in the reflectance of different bands is more gentle than at the jointing stage. Specifically, the variation in the reflectance of visible bands is slightly slow within the first 100 m, and the variation in the reflectance of NIR and red-edge bands is weakened (Figure 2C). At the milking stage, the reflectance of each band changes relatively steadily (Figure 2D).

#### The variation of vegetation indices with UAV flying altitudes

**FIGURE 3:**
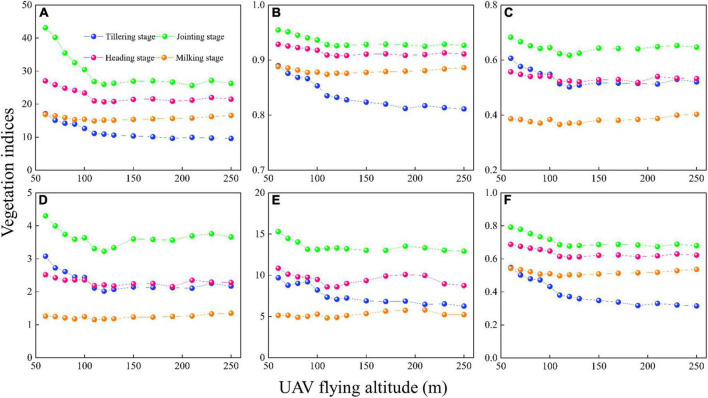
Variation in VIs with different UAV-FAs: **(A)** RVI; **(B)** NDVI; **(C)** NDRE; **(D)** CI_red edge_; **(E)** CI_green_; and **(F)** WDRVI.

The changes in VIs with UAV-FAs in different periods are shown in Figure 3. It can be seen that almost all VIs show similar trends in different periods. At the tillering, jointing, and heading stages, VIs change sharply within 100 m with UAV-FAs, but become stable after 100 m. This is consistent with the variation in the reflectance of the visible bands with UAV-FAs. In addition, VIs at the milking stage are nearly unaffected by UAV-FAs. With the growth of rice, the variation in VIs gradually weakens within the first 100 m, that is, the most significant changes occur at tillering and jointing stages, then weaken significantly at the heading stage, and remain almost unchanged at the milking stage.

#### Analysis of contributing factors to reflectance variation with UAV flying altitudes

**FIGURE 4:**
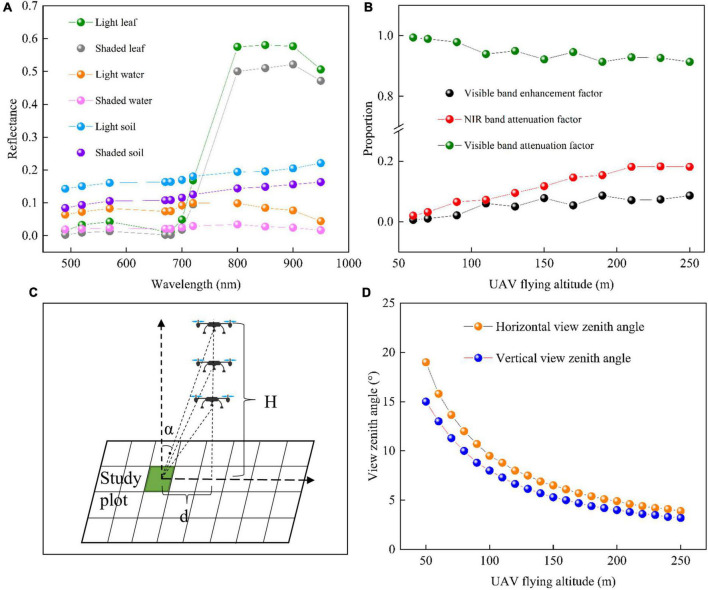
The changes caused by different UAV-FAs: **(A)** Reflectance of different endmembers in the rice field; **(B)** mixed pixel decomposition results; **(C)** schematic diagram of UAV observation angle change at different altitudes, α is the VZA and H is the UAV flying altitude; and **(D)** the changes in edge-plot FOV with the increase of UAV-FAs.

The FCLS-LSM model was used to analyze the reasons for the changes in reflectance obtained from the same area observed at different altitudes. Taking the edge plot at the jointing stage as an example, after six kinds of endmembers (light leaf, shaded leaf, light water, shaded water, light soil, and shaded soil) were selected and measured, the spectral curve of each endmember (Figure 4A) was obtained by convolution of the spectral response function of Mini-MCA12 camera. As shown in Figure 4A, in the NIR bands, the reflectance of rice was much higher than that of the background, but the opposite was true in the visible bands. The abundances (i.e., proportions) of different endmembers were acquired through unmixing, and the reflectance changes were described through the combinations of different endmembers (Figure 4B). The NIR band attenuation factor was defined as the sum of all background abundances (light water + shaded water + light soil + shaded soil), and the visible band enhancement factor was calculated by the combination of background abundances (light water + light soil + shaded soil – shaded water). The results in Figure 4B demonstrate that below 100 m, the visible band enhancement factor gradually increases with UAV-FAs, after which it remains stable. The NIR band attenuation factor increases with UAV-FAs until it changes slowly after 170 m. This shows no difference with the variation trend of NIR-band reflectance at the jointing stage (Figure 2B). Thus, as the background ratio increases, the reflectance of the NIR bands decreases, while the reflectance of the visible bands increases.

The observation angles of the same rice plot at different UAV-FAs are shown in Figure 4C. It can be seen that with the increase of UAV-FAs, the view zenith angle (VZA) of the same observation area gradually decreases. At a certain flight altitude, the edge-plot VZA of the image is equal to half of the FOV, so the distance from the study plot to the UAV in the vertical direction (d) can be calculated, and the variation in VZA at different UAV-FAs can be simulated according to the changing flight altitudes (H). The results in Figure 4D indicate that when the flight altitude rises from 50 to 100 m, the VZA drops sharply, and the trend gradually slows down after 100 m. The plot in this study is not located at the extreme edge of the image. Hence, the VZA at 100 m will shift to the right in Figure 4D, which is close to the stable state. At this point, the observation is closest to the orthotopic position.

Thus, observations at different altitudes are observations at different angles. The different proportions of the rice canopy and background observed at different angles, as well as the differences in the proportion of light and shaded leaves in the canopy, make the observed reflectance of the rice plot show the characteristics of bidirectional reflection.

### Impact of different radiometric calibration methods and UAV flying altitudes on rice growth monitoring

Different RCMs will produce large differences in reflectance and VIs (Figure 1 and Table 4). Thus, it is necessary to evaluate the impact of such differences in rice growth monitoring to guide practical applications. The EL-based reflectance often appears as outliers in the red bands, and the derived VIs will exceed the normal range (e.g., NDVI greater than 1). CI_green_ without red bands was selected to analyze the impact of different RCMs. To avoid the influence of bidirectional reflection, the 100 m images in Test 1 and Test 2 were used as the base map for calculating the CI_green_. The linear regression models for estimating LAI, AGB, and CCC of rice are shown in Table 5. It turns out that the PEL performs better than EL and SEL methods in Test 1 and Test 2. In Test 1, the differences in LAI, AGB, and CCC prediction errors based on three RCMs are approximately 8, 2, and 10%, respectively. In Test 2, these differences are about 2%. In general, the PEL method has a stable advantage in rice growth monitoring.

**TABLE 5:** The linear regression models for monitoring rice growth parameters based on three RCMs in Test 1 and Test 2.

Experimental name	RCMs	Regression equation	*R* ^2^	RMSE	RRMSE%
Test 1	EL	LAI = 0.2138 × CI_green_ + 2.1213	0.6159	1.1894	24.43
	SEL	LAI = 0.3392 × CI_green_ + 1.2471	0.7714	0.9175	18.84
	PEL	LAI = 0.3360 × CI_green_ + 1.0176	**0.8364**	**0.7772**	**15.96**
	EL	AGB = 31.8780 × CI_green_ + 58.2600	0.6283	258.5627	47.60
	SEL	AGB = 38.2660 × CI_green_ + 46.7340	0.5478	267.8949	49.32
	PEL	AGB = 39.6540 × CI_green_ + 3.7036	**0.6534**	**255.5819**	**47.05**
	EL	CCC = 8.7479 × CI_green_ + 96.1880	0.5419	56.6725	27.16
	SEL	CCC = 14.7750 × CI_green_ + 50.8720	0.7689	40.2487	19.29
	PEL	CCC = 14.3360 × CI_green_ + 44.2940	**0.8001**	**37.4370**	**17.94**
Test 2	EL	LAI = 0.2944 × CI_green_ + 0.6926	0.8350	0.8195	18.38
	SEL	LAI = 0.3210 × CI_green_ + 0.6811	0.8026	0.8755	19.63
	PEL	LAI = 0.3390 × CI_green_ + 0.4682	**0.8533**	**0.7820**	**17.53**
	EL	AGB = 42.1860 × CI_green_ + 30.6610	0.4654	271.4445	48.08
	SEL	AGB = 45.0950 × CI_green_ + 31.0940	0.4403	277.7335	49.20
	PEL	AGB = 47.5830 × CI_green_ + 4.9777	**0.4690**	**270.5139**	**47.92**
	EL	CCC = 11.9540 × CI_green_ + 28.8210	0.8588	30.9864	17.05
	SEL	CCC = 13.2190 × CI_green_ + 26.1690	0.8492	31.5024	17.33
	PEL	CCC = 13.9210 × CI_green_ + 17.8800	**0.8975**	**27.5072**	**15.13**

The method with the best performance is represented by bold values.

As for the impact of different UAV-FAs on rice growth monitoring, the UAV-based images were processed by the PEL method. The results in Table 6 demonstrate that the differences in prediction results of rice growth parameters caused by UAV-FAs were more pronounced in Test 2 (50 and 100 m). In Test 1 (100–250 m), the differences in LAI, AGB, and CCC prediction errors based on different UAV-FAs were approximately 1, 2, and 1%, respectively. While in Test 2, these differences were about 8, 2, and 3%, respectively. The difference in LAI estimating results was the most significant.

**TABLE 6:** The linear regression models for monitoring rice growth parameters based on different UAV-FAs in Test 1 and Test 2.

Experimental name	UAV-FAs (m)	Regression equation	*R* ^2^	RMSE	RRMSE%
Test 1	100	LAI = 0.3360 × CI_green_ + 1.0176	0.8364	0.7762	15.94
	150	LAI = 0.3539 × CI_green_ + 0.9984	0.8546	0.7318	15.03
	200	LAI = 0.3421 × CI_green_ + 0.9721	0.8317	0.7874	16.17
	250	LAI = 0.3554 × CI_green_ + 0.8772	0.8411	0.7649	15.71
	100	AGB = 39.6540 × CI_green_ + 3.7036	0.6534	162.6172	36.55
	150	AGB = 43.5090 × CI_green_ – 4.7125	0.6363	165.2271	36.81
	200	AGB = 38.6880 × CI_green_ + 6.2879	0.6133	171.7673	38.60
	250	AGB = 40.1870 × CI_green_ + 1.6108	0.6130	171.8381	38.62
	100	CCC = 14.3360 × CI_green_ + 44.2940	0.8001	37.4370	17.94
	150	CCC = 14.6560 × CI_green_ + 48.3090	0.8103	35.0322	17.07
	200	CCC = 15.0250 × CI_green_ + 37.4670	0.8430	33.1784	15.90
	250	CCC = 15.5450 × CI_green_ + 34.0210	0.8455	32.9088	15.77
Test 2	50	LAI = 0.2788 × CI_green_ + 0.8889	0.7761	1.1150	25.00
	100	LAI = 0.3390 × CI_green_ + 0.4682	0.8533	0.7820	17.53
	50	AGB = 39.5530 × CI_green_ + 55.1480	0.4301	280.2471	49.64
	100	AGB = 47.5830 × CI_green_ + 4.9777	0.4690	270.5139	47.92
	50	CCC = 11.4960 × CI_green_ + 34.5720	0.8228	33.9458	18.67
	100	CCC = 13.9210 × CI_green_ + 17.8800	0.8975	27.5072	15.13

### Impact of different radiometric calibration methods and UAV flying altitudes on yield prediction

**FIGURE 5:**
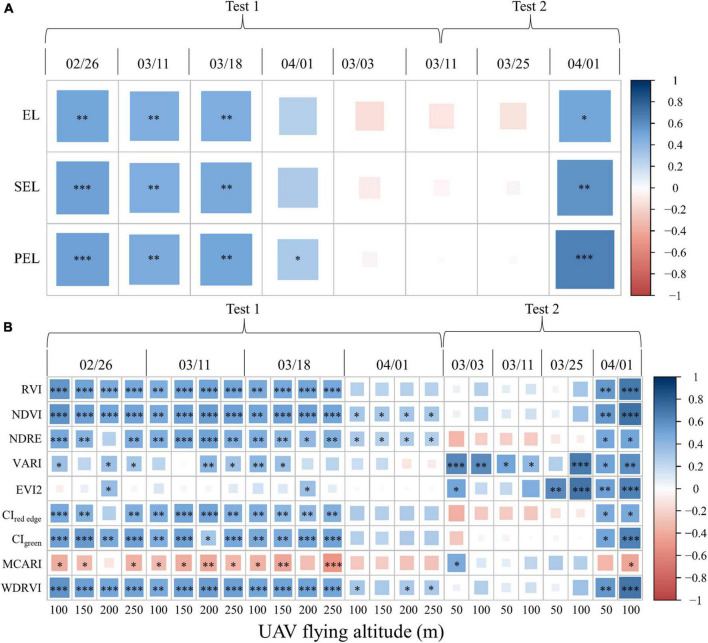
Impact of different RCMs and UAV-FAs on yield prediction: **(A)** Pearson correlation coefficient between rice yield and CI_green_ based on three RCMs in Test 1 and Test 2; **(B)** Pearson correlation coefficient between rice yield and VIs at different UAV-FAs in Test 1 and Test 2 (^***^, ^**^, and * represent the significant correlation at the 0.001, 0.01, and 0.05 levels, respectively).

When analyzing the influence of different RCMs on yield prediction in a single stage, the images derived from an altitude of 100 m in Test 1 and Test 2 were selected. Moreover, the CI_green_ was also utilized to correlate with the yield of different stages. Quantitative evidence shown in Figure 5A indicates that the PEL reveals a better correlation with the yield at each stage in Test 1 and Test 2, particularly at the heading stage (significance changes).

The Pearson correlation coefficient between rice yield and PEL-based VIs at different UAV-FAs in Test 1 and Test 2 are shown in Figure 5. The darker and larger the colored rectangles in the heat map, the stronger the correlation. It can be found that, as a whole, there was little difference in the correlation between VIs and yield at different altitudes in Test 1, but there was a significant difference in Test 2 at each stage. Indices with the strongest correlation with the rice yield at booting (EVI2) and heading stages (WDRVI) were selected for quantitative comparison. The linear regression models for predicting rice yield using PEL-based VIs at booting and heading stages in Test 2 are shown in Table 7. It can be seen that the difference in yield prediction errors based on 50 and 100 m altitudes is about 2%.

**TABLE 7:** The linear regression models for predicting rice yield at booting and heading stages in Test 2.

Growth stage	UAV flying altitude (m)	Regression equation	*R* ^2^	RMSE	RRMSE%
Booting stage (03/25)	50	Yield = 3186.2660 × EVI2 - 2002.0312	0.3859	124.7462	15.4756
	100	Yield = 5475.6664 × EVI2 - 3455.1211	0.5177	110.5548	13.7151
Heading stage (04/01)	50	Yield = 1337.1689 × WDRVI - 120.3483	0.3165	131.6050	16.3265
	100	Yield = 2867.1955 × WDRVI - 1007.9788	0.5170	110.6357	13.7251

**FIGURE 6:**
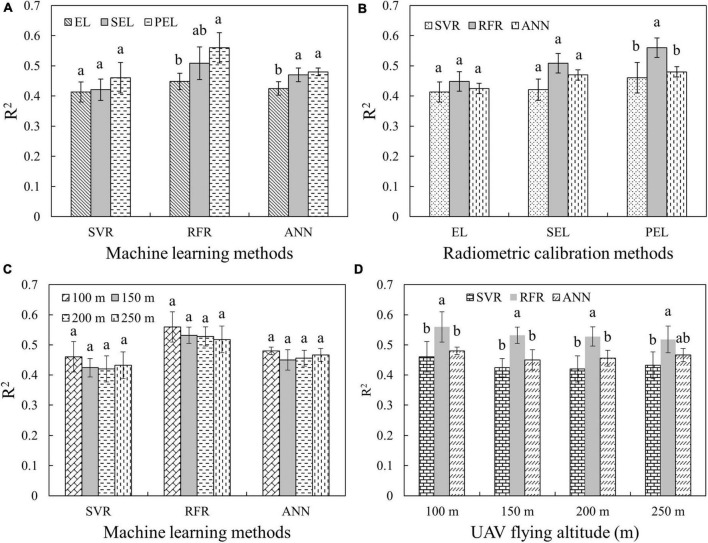
*R*^2^-values of multi-stage rice yield prediction using machine learning methods with different RCMs or UAV-FAs: **(A)** Comparison of different RCMs and same machine learning methods using CI_green_; **(B)** comparison of same RCMs and different machine learning methods using CI_green_; **(C)** comparison of different RCMs and same UAV-FAs using NDVI; and **(D)** comparison of same UAV-FAs and different machine learning methods using NDVI.

As for the yield estimation of multi-variety rice, VIs in the whole period in Test 1 was used for yield prediction by the machine learning methods (SVM, RFR, and ANN) with 10-fold cross-validation because the relationship between VIs and yield was not directly linear. Each model was run 10 times as a result group, and an analysis of variance (ANOVA) was conducted to analyze the differences among individual groups. CI_green_ and NDVI are employed in the differential analysis of RCMs and UAV-FAs, respectively. As shown in Figure 6, the averaged value of *R*^2^ with the same lowercase letters (a, b, and c) is not significantly different by Tukey’s test at a significance level of 5%. It turns out that the SEL- and PEL-based yield estimation accuracy is significantly higher than that of EL using RFR and ANN. The three RCMs share little difference when using SVM (Figure 6A). The RFR is proved to be the best predictor using the PEL method, and the performance of the three machine learning methods share little difference using EL and SEL methods (Figure 6B). The results in Figure 6C demonstrate that the altitude above 100 m has no significant effect on yield prediction. However, there are some differences between the results of different yield estimation methods (Figure 6D). The RFR has the highest prediction accuracy in multi-period rice yield estimation at 100–250 m altitude. Consequently, different RCMs and UAV-FAs have some impacts on single- and multi-period rice yield estimation.

## Discussion

### The comparison and selection of radiometric calibration methods

In precision agriculture, the selection of RCMs and the evaluation of reflectance accuracy are often ignored (Jiang et al., 2019; Rosas et al., 2020; Wan et al., 2020). The results presented in Figure 1 and Tables 2, 3 show that the EL method is suitable for the conversion of high reflectance (e.g., the slabstone and the rice at the tillering stage), while the SEL method can obtain more accurate low reflectance (e.g., the grassland and the rice at jointing, booting, and heading stages). Therefore, the PEL method was proposed to combine the advantages of EL and SEL and to automatically obtain high-precision reflectance for different ground objects using multiple calibration panels. In the visible bands, SEL is useful for the low reflectance of the vegetation itself. However, when plants are mixed with high reflectance background (such as soil), the performance of SEL is worse than that of EL. When vegetation almost covered the background (e.g., rice and grassland with a closed canopy), the ability of SEL to obtain low reflectance is highlighted, while the PEL can automatically perform pixel-to-pixel reflectance conversion and obtain high-precision vegetation and background reflectance. This paves the way for analyzing the reflectance changes at different UAV-FAs.

The existing study shows that the accuracy of VIs derived from different sensors does not directly depend on the accuracy of reflectance (Deng et al., 2018b). That is to say, for different sensors, when the accuracy of reflectance is higher, it is not necessary that the accuracy of VIs should also be higher. The reasons are as follows: (i) the accuracy of reflectance of each band is different, (ii) bandwidths or central bands are different, and (iii) the reflectance is affected by the weather. However, in this study, the accuracy of VIs of the same sensor depends on its accuracy of reflectance. The results presented in Table 4 demonstrate that SEL- and PEL-based VIs (except CI_green_ in Test 4) are more accurate than those based on EL in Test 1, Test 2, and Test 4. The accuracy of EL-based CI_green_ is higher than that of SEL in Test 4 due to the accuracy of the reflectance. The reflectance of 550, 570, and 700 nm of grassland in Test 4 has a higher accuracy based on EL (PEL) than SEL.

### The response of reflectance and vegetation indices to different UAV flying altitudes

The vegetation canopy reflectance shows anisotropic characteristics with the change of incidence and observation angles, which is generally described by the bidirectional reflection distribution function (BRDF) (Roy et al., 2016). The BRDF characteristics of vegetation canopy mainly depend on the following factors: (i) the optical characteristics of leaves and ground background; and (ii) canopy structure characteristics, including LAI, leaf inclination, canopy geometry, density, and distribution (Qiu et al., 2021). The changes in rice canopy reflectance with UAV-FAs at the same stage are mainly caused by the difference in the observation angles (Figure 6C).

**FIGURE 7:**
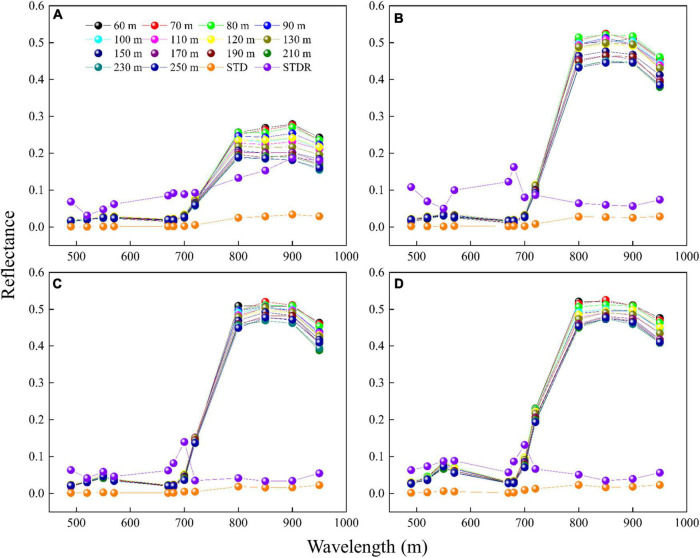
Reflectance spectral curves of rice at different altitudes and stages (STD represents the standard deviation of spectral reflectance at all altitudes and STDR is the ratio of the STD to the reflectance at 250 m): **(A)** Tillering stage; **(B)** jointing stage; **(C)** heading stage; **(D)** milking stage.

The reflectance of rice at different altitudes and growth stages was obtained with high accuracy using PEL methods. The results in Figure 2 show that the reflectance variation in the different bands of rice at diverse UAV-FAs shares significant differences at different growth durations. To analyze the sensitivity of different bands to UAV-FAs, the reflectance curves of rice at the altitudes of 60–250 m in different periods are shown in Figure 7. The sensitivity is expressed as the ratio of the standard deviation (STD) of spectral reflectance at all altitudes to the reflectance (STDR) in the orthophoto direction (approximately substituted by the reflectance at 250 m). It can be seen that the variation range and sensitivity (STDR) of reflectance in NIR bands are more obvious than that in visible bands at the tillering stage (Figure 7A). This was due to the effect of water on the tillering stage, resulting in reduced sensitivity of visible bands (Gatebe and King, 2016). At the jointing, heading, and milking stages, the reflectance variation range of NIR bands is greater than that of visible bands, but the sensitivity is less than that of visible bands (Figures 7A–D). At these stages, the anisotropy effect of reflectance is the strongest in the red band (STDR > 0.1). In the NIR bands, the leaf absorption of vegetation is weak, and the reflectance and transmittance are high, which makes the multiple scattering effects inside the canopy stronger and reduces the anisotropy of vegetation in these bands, while the strong absorption of chlorophyll makes the anisotropy stronger in the red bands (Sandmeier et al., 1998). Therefore, the change of reflectance is distinctly weakened at the heading and milking stages because the appearance of the panicle makes the canopy not easy to be penetrated by light.

The FCLS-LSM model was used to analyze the changes in the components of multispectral images of paddy fields at the jointing stage. The results in Figure 4B showed that the fluctuations in the proportion of rice leaves and soil background, and the ratio of light and shaded leaves in the canopy are the reasons for the changes in canopy reflectance. The enhancement and attenuation factors in the visible bands change significantly before 100 m and then tend to be stable, while the NIR band attenuation factors keep unchanged after 170 m, because in the NIR bands, the reflectance of rice is much greater than that of the background (Figure 4A). In addition, the NIR bands have strong penetration, and the reflectance will decrease significantly as long as the background increases slightly. In the visible bands, the reflectance gap between the background and rice is relatively small, and the weak change of the background proportion will not lead to a drastic response of the reflectance. It can also be found in Figure 4D that after 100 m, the VZA variation of the plot at the edge of the image gradually becomes flat with the change of altitudes, which also proves that there is little fluctuation in the different components of the multispectral images after 100 m, and the edge plot at this altitude is close to the orthophoto direction. Therefore, the variation in the reflectance of different bands with UAV-FAs is related to the reflectance of the backgrounds.

The fluctuation in the reflectance will cause a change in VIs. It is found in Figure 3 that the variation trend of VIs and reflectance of visible and red-edge bands in different periods are consistent, appearing that the VIs change significantly within 100 m at tillering, jointing, and heading stages, and remain unchanged beyond 100 m. The main reason for this phenomenon is that the reflectance of NIR bands is generally taken as the numerator in the calculation of VIs, while the reflectance of visible and red-edge bands can be taken as the denominator. Therefore, small changes in the denominator will eventually be amplified, and the influence of numerator changes will be much smaller.

### Effects of radiometric calibration methods and UAV flying altitudes on the accurate acquisition of rice phenotypes

The VIs derived from the combination of reflectance in different bands have proven to be a good indicator to monitor crop growth and predict the yield (Deng et al., 2018b; Hassan et al., 2019; Luo et al., 2020). For crop growth monitoring (like LAI, AGB, and chlorophyll content), a multi-stage model is acceptable (Li et al., 2020). Both single-period and multi-period models are useful for yield estimation (Gong et al., 2018; Wang et al., 2019; Wan et al., 2020; Duan et al., 2021). Machine learning is the most widely used method in multi-period crop yield prediction. In this study, rice growth monitoring (multi-stage) and yield prediction (single and multi-stage) were carried out to verify and evaluate the effects of RCMs and UAV-FAs.

**FIGURE 8:**
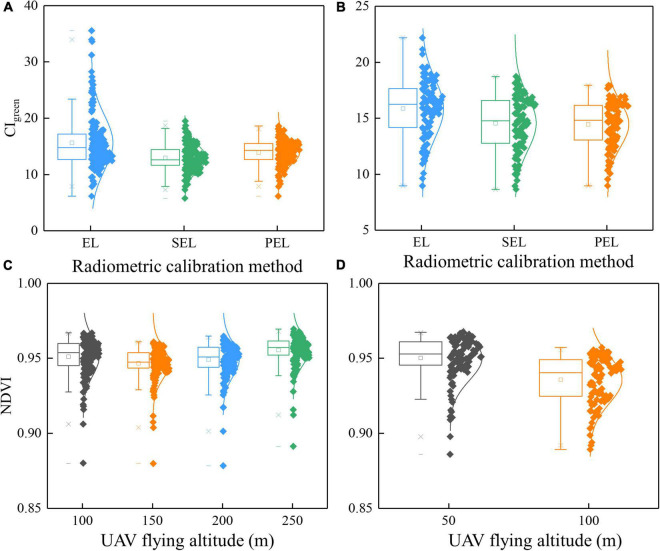
Boxplot for the comparison of VIs based on different RCMs and UAV-FAs: **(A)** CI_green_ based on different RCMs in Test 1; **(B)** CI_green_ based on different RCMs in Test 2; **(C)** NDVI based on different UAV-FAs in Test 1; **(D)** NDVI based on different UAV-FAs in Test 2.

The results in Table 5 and Figure 5A indicate that the PEL method holds the highest accuracy in LAI, AGB, CCC estimation, and single-stage yield prediction. The comparison of CI_green_ based on the three RCMs is shown in Figures 8A,B. It can be seen that the EL-based CI_green_ is different from the others (including range and distribution). Hence, the selection of appropriate RCMs has a very notable impact on precision agriculture (Deng et al., 2018b).

The results of different UAV-FAs presented in Tables 6, 7 and Figure 5 show that the altitude change above 100 m has no conspicuous impact on growth parameter estimation and yield prediction. The variables based on 50 and 100 m have a great influence on single-period yield prediction and have a relatively weak impact on growth simulation. Of course, the size and number of plots in Test 2 are less than those in Test 1. This reduces the impact of altitude to a certain extent because the plots in Test 2 are closer to the center of the image. The comparison of NDVI based on different UAV-FAs is shown in Figures 8C,D. The range and shape of NDVI based on 100–250 m are similar, and the difference between 50 and 100 m is prominent. At low altitudes, NDVI is larger and more easily saturated, which reduces the accuracy of the estimation model.

It is also crucial to select different machine learning methods for yield estimation with multi-period variables. For example, SVM can cover up the difference in yield estimation caused by different RCMs, while RFR and ANN can show the superiority of SEL and PEL (Figure 6A). The impact of different RCMs is also reflected in whether the advantages of machine learning methods can be highlighted: there is no significant difference in the estimation accuracy of the three machine learning methods when EL and SEL are used, while the accuracy of RFR is significantly higher than that of other methods when PEL is used (Figure 6B). Therefore, RCMs and machine learning methods have a mutual influence on yield estimation results, which should be paid attention to in the selection. The altitude change above 100 m does not have a significant impact on the multi-period yield estimation results (Figure 6C). The yield estimation accuracy of RFR is the highest, and there is no significant difference between SVR and ANN (Figure 6D).

Compared with the vertical downward observation at high altitude, the vegetation directional reflectance obtained by RS at low altitude contains abundant information on vegetation canopy structure. Therefore, more attention should be paid to the extraction of crop phenotype information from low-altitude images, particularly for sensors with large FOV.

## Conclusion

Multispectral reflectance can be affected by RCMs and UAV-FAs. In this paper, the reflectance derived from different RCMs was compared, and accurate reflectance at different altitudes was obtained. It was found that the EL and SEL methods performed well in the prediction of high reflectance and low reflectance, respectively. The PEL method combining the advantages of EL and SEL showed the highest accuracy in rice growth monitoring (LAI, AGB, and CCC estimation) and yield prediction. In addition, the selection of machine learning methods would have a certain impact on multi-period rice yield estimation. Due to the differences in observation angles caused by UAV-FAs, the proportion changes of light and shaded rice and background made the reflectance fluctuate at different altitudes, which was apparent at tillering and jointing stages, and weakened at heading and milking stages. Likewise, VIs also showed certain variation rules, changing violently within 100 m and then remaining stable. The experimental data showed that the results of rice growth monitoring and yield prediction (using single and multi-period variables) differed significantly at different low altitudes (50 and 100 m) and shared little difference at high altitudes (100, 150, 200, and 250 m). The specific altitude value is determined by the FOV of the sensor and the characteristic of the ground object. In future work, more attention will be paid to the acquisition of crop phenotype information from low-altitude multispectral images as a result of the inclusion of more canopy structure information.

## Data availability statement

The original contributions presented in this study are included in the article/supplementary material, further inquiries can be directed to the corresponding author.

## Author contributions

SL wrote the manuscript. SF provided the study ideas and completed the experimental design. XJ provided suggestions and edited the manuscript. KY, YL, and SL measured the experimental data. YL and SL conducted the UAV flights. SF and XJ provided comments on the revision of the manuscript. All authors read and approved the final manuscript.
